# Interdisciplinary diabetes care teams operating on the interface between primary and specialty care are associated with improved outcomes of care: findings from the Leuven Diabetes Project

**DOI:** 10.1186/1472-6963-9-179

**Published:** 2009-10-07

**Authors:** Liesbeth Borgermans, Geert Goderis, Carine Van Den Broeke, Geert Verbeke, An Carbonez, Anna Ivanova, Chantal Mathieu, Bert Aertgeerts, Jan Heyrman, Richard Grol

**Affiliations:** 1 Department of General Practice, Katholieke Universiteit Leuven, Leuven, Belgium; 2 Biostatistal Centre, Katholieke Universiteit Leuven, Leuven, Belgium; 3 Leuven Statistics Centre, Katholieke Universiteit Leuven, Leuven, Belgium; 4 Endocrinology, University Hospitals Leuven, Leuven, Belgium; 5 Scientific Institute for the Quality of Healthcare, Radboud University Nijmegen, the Netherlands

## Abstract

**Background:**

Type 2 diabetes mellitus is a complex, progressive disease which requires a variety of quality improvement strategies. Limited information is available on the feasibility and effectiveness of interdisciplinary diabetes care teams (IDCT) operating on the interface between primary and specialty care. A first study hypothesis was that the implementation of an IDCT is feasible in a health care setting with limited tradition in shared care. A second hypothesis was that patients who make use of an IDCT would have significantly better outcomes compared to non-users of the IDCT after an 18-month intervention period. A third hypothesis was that patients who used the IDCT in an Advanced quality Improvement Program (AQIP) would have significantly better outcomes compared to users of a Usual Quality Improvement Program (UQIP).

**Methods:**

This investigation comprised a two-arm cluster randomized trial conducted in a primary care setting in Belgium. Primary care physicians (PCPs, n = 120) and their patients with type 2 diabetes mellitus (n = 2495) were included and subjects were randomly assigned to the intervention arms. The IDCT acted as a cornerstone to both the intervention arms, but the number, type and intensity of IDCT related interventions varied depending upon the intervention arm.

**Results:**

Final registration included 67 PCPs and 1577 patients in the AQIP and 53 PCPs and 918 patients in the UQIP. 84% of the PCPs made use of the IDCT. The expected participation rate in patients (30%) was not attained, with 12,5% of the patients using the IDCT. When comparing users and non-users of the IDCT (irrespective of the intervention arm) and after 18 months of intervention the use of the IDCT was significantly associated with improvements in HbA1c, LDL-cholesterol, an increase in statins and anti-platelet therapy as well as the number of targets that were reached. When comparing users of the IDCT in the two intervention arms no significant differences were noted, except for anti-platelet therapy.

**Conclusion:**

IDCT's operating on the interface between primary and specialty care are associated with improved outcomes of care. More research is required on what team and program characteristics contribute to improvements in diabetes care.

**Trial registration:**

NTR 1369.

## Background

Despite its multi-system effects, diabetes is a controllable disease, and there is unequivocal evidence that early and proactive, continuous monitoring and treatment can significantly reduce its human and economic toll [1-3]. Many guidelines provide targets that are desirable for most patients with diabetes[4]. Literature demonstrates however that many patients with type 2 diabetes mellitus (DM) still don't receive the care they need[5], as physicians overrate the quality of the care they already deliver and substantially underestimate the number of patients in need of intensified pharmacotherapy and lifestyle interventions[6]. We therefore need quality improvement programs that promote comprehensive and proactive diabetes care at different levels of the health care system. Components of such care that many physicians find difficult to provide include risk factor reduction, periodic examination for early signs of complications, ongoing education and behavioral interventions and health promotion[7]. The failure to initiate or to intensify therapy in a patient not at evidence-based treatment targets is defined as 'clinical inertia'[8]. Besides physician factors the principal sources for clinical inertia include patient factors (e.g. resistance to adopting lifestyles that support optimal disease care) and organizational factors (e.g. a lack of a team approach to care)[9,10]. These three sources interact in complex ways and quality improvement interventions to reduce clinical inertia therefore need to be multifactorial in nature[11]. A multifactorial approach to chronic care delivery is advocated by multiple authors that have developed conceptual models for this purpose [12-14]. A widely used conceptual model for improving chronic illness care is the Chronic Care Model (CCM)[15]. Despite a diabetes prevalence rate of 7.9% in Europe[16], very few countries in Europe have national or even local programs that target this chronic condition building on multiple dimensions of the CCM. Previous studies have shown that it is not the intensity of the program, i.e. the number dimensions of the CCM that are applied which determines outcomes of care[17]. Important predictors of success are the type of interventions and the implementation strategies applied[18]. One such important component of quality improvement programs in diabetes care are considered interdisciplinary diabetes care teams[19]. These teams often focus on clinical guidance to primary care physicians and the provision of a broad range of educational services to patients[20]. There is a particular paucity of evidence with regard to the effectiveness of interdisciplinary diabetes care teams operating on the interface between primary and specialty care[21]. This interface refers to collaborative relationships between primary and specialty care aimed at improvements in efficiency and effectiveness of care. This study reports on the effectiveness of primary care-based interdisciplinary diabetes care teams that are actively guided and supported by a specialist team from secondary care. The teams are embedded in a two-arm multifaceted quality improvement program targeting adherence to guidelines and clinical inertia in primary care physicians.

A first study hypothesis was that the implementation of an interdisciplinary diabetes care team (IDCT) in support of primary care physicians is feasible in a health care setting with limited tradition in shared care. A second hypothesis was that patients who make use of an IDCT would have significantly better outcomes compared to non-users of the IDCT (irrespective of the intervention arm) and after an 18-month intervention period. A third hypothesis is that patients who used the IDCT in an advanced quality improvement program (AQIP) would have better outcomes compared to users in a usual quality improvement program (UQIP).

This study (The Leuven Diabetes Project) is intended to create the basis for the development of a national diabetes care program that set out national standards for the care of people with diabetes with the aim to raise the quality of services and to promote interdisciplinary shared care.

## Methods

The study protocol of our trial has been peer-reviewed and published previously[22]. We summarize information on the study design, participants and key interventions of the IDCT. For detailed information on the sample size, randomization, allocation concealment, method for data collection and detailed descriptions on the interventions provided we refer to our study protocol and the CONSORT Checklist of items of cluster randomized trials[23], as presented in Table 1.

**Table 1:** CONSORT Checklist of items of cluster randomized trials.

**Paper section and topic**	**Item**	**Descriptor**
**Title and abstract** Design	1	→ Interdisciplinary Diabetes Care Teams operating on the interface between primary and specialty care are associated with improved outcomes of care: Findings from the Leuven Diabetes Project.

**Introduction** Background	2	→ Scientific background and explanation of rationale: see Background section → Clustering: randomization at GP-level, primary/secondary outcomes at patient level. → Randomized per practice; stratified

**Methods** Participants	3	→ All 379 primary care physicians (PCP's) that actively execute their profession in the project region were invited to participate. → The only inclusion criterion for the PCP's is the agreement to bring in all their known patients with type 2 diabetes mellitus. In this way selection bias is prevented. Patients had to provide informed consent before their data could be transmitted for collection and analysis. Only patients with type 2 diabetes mellitus were be included in the study, regardless of their age. Patients who were not capable to provide informed consent were excluded from the study. → Data were collected on paper files and from medical records

Interventions	4	→ See methods section

Objectives	5	→ See methods section

Outcomes	6	→ The primary endpoints of the study were the proportion of patients reaching three clinical ADA-targets: (1) HbA1c < 7%; (2) SBD ≤ 130 mm Hg; (3) LDL-C < 100 mg/dl. Secondary endpoints were the mean improvements in individual parameters of 12 validated parameters, i.e. HbA1c, LDL-C, HDL-C, Total Cholesterol, SBP, Diastolic Blood Pressure (DBP), weight, physical exercise, healthy diet, smoking status, statin and anti-platelet therapy.

Sample size	7	→ The financer to the project imposes a sample size of minimal one third of the potential PCP's. Using the calculator of the university of Aberdeen, sample size for cluster trials was computed. With a significance level of 0.05 and assumed Intra Cluster Coefficient of 0.1, we calculated that 114 clusters with a cluster size of 20 gave 80% power to detect between AQIP and UQIP a 10% in the absolute difference in the proportion of patients achieving a 10% improvement in the primary biochemical endpoints. Based on the fitted mixed models the observed ICC values are: HBA1C: 0.0445, SBD: 0.0466, LDL Cholesterol: 0.0399.

Randomization Sequence generation	8	→ After the recruitment period, using computer-generated numbers, a researcher not involved the study and blind to the identity of the practices will perform a randomization stratified by practice size (solo/duo/group practice) and the presence/absence of an electronic medical recording system.

Allocation concealment	9	→ Program Manager - invitation, stratified. → To minimize the possibility of selection bias all patients within a cluster were included

Implementation	10	→ Allocation: Van Den Broeke Carine, researcher to the scientific team, → Enrollment: Borgermans Liesbeth, researcher to the scientific team, → Assignment: program manager

Blinding (masking)	11	→ No blinding was possible at physician level, (both groups presented as 'intervention'), but patients didn't know to which intervention arm their physician belonged.

Statistical methods	12	→ See methods section, sub-heading statistical analysis.

### Study design

The study was a cluster-randomized trial with before/after measurements and two intervention arms. A cluster design was necessary since randomization was performed on a practice level, the intervention happened on the physician level, but a large part of the data were analyzed at the patient level. The implementation period of the trial was 18 months.

### Participants

All 336 active primary care physicians (PCPs) in the project region were invited to participate in the project. These PCPs work in a semi-rural setting with 357.000 inhabitants and serve predominantly Caucasian patients with type 2 diabetes mellitus. Primary care physicians provide care for approximately 80% of patients with type 2 diabetes, and are often the sole providers of care who have no or limited experience with interdisciplinary shared care. A fixed fee of 60 Euro was issued per registered patient. The only inclusion criterion for the PCPs was agreeing to recruit all patients with type 2 diabetes mellitus to prevent selection bias. Patients with diabetes were identified using electronic searching in computerized records and laboratory lists of patients with increased glycaemia or registered HbA1c. Diabetes was defined in accordance with the 2003 ADA criteria[24] with PCPs making the final diagnosis. Patients with type 1 DM and those patients who could not provide informed consent were excluded from the study. All patients were blinded to the study design, but PCPs were not, as they were involved in the execution of the programs. As a general referral indication, PCPs were recommended to refer to the IDCT in case treatment targets were not met, despite own efforts. Patients only had access to the IDCT after referral of their PCP. The interventions of the IDCT were free of charge for all included patients.

### Intervention

The UK Medical Research Council (MRC) Framework[25] for the development and evaluation of complex interventions for randomized control trials (RCT) was used as a theoretical guide to designing the intervention. Two separate groups were defined: the first group received a Usual Quality Improvement Program (UQIP-program) and a second group received an Advanced Quality Improvement Program (AQIP-program). A summary of the differences in interventions between the AQIP and the UQIP is presented in Table 2, 3 and 4. Briefly summarized, the UQIP arm aimed to improve adherence to evidence-based guidelines and to reduce the rate of clinical inertia in primary care physicians (PCPs). The term 'usual' was applied since these interventions represent standard requirements for improving the quality of diabetes care in most health care systems[26]. The AQIP arm received identical interventions, but also included supplementary interventions that extensively focused on behavior changes in patients and providers.

**Table 2:** Interventions for UQIP versus AQIP (patient).

**PATIENT**		
	**USUAL QUALITY IMPROVEMENT PROGRAM (UQIP)**	**ADVANCED QUALITY IMPROVEMENT PROGRAM (AQIP)**

**Patient education**	Medical assessments and education upon referral of the PCPs by diabetologist or Diabetes Care Team = internist, nurse educator, dietician and ophthalmologist	Medical assessments and education upon referral of the PCPs by diabetologist or Diabetes Care Team = internist, nurse educator, flying educator, dietician, ophthalmologist and health psychologist

**Promotion of self-management**	----	Education of patients in practice (by flying educator)
	
	----	Education at patient's home (by flying educator)
	
	----	Counseling by health psychologist
	
	----	Structured educational materials from IDCT

	----	Structured educational materials from community organizations
	
	----	Group educational sessions for patients and family members
	
	----	Free access to blood monitoring tools for self-management

**Table 3:** Interventions for UQIP versus AQIP (professional).

**PROFESSIONAL**		
	**USUAL QUALITY IMPROVEMENT PROGRAM (UQIP)**	**ADVANCED QUALITY IMPROVEMENT PROGRAM (AQIP)**

**Clinician education**	Distribution of treatment protocol	Distribution of treatment protocol

	Two post-graduate educational sessions	Four post-graduate educational sessions provided by diabetologist (opinion leader):
	- Evidence based guidelines	Evidence-based guidelines and principles of shared care
	- The use of insulin	The use of insulin
		Patient-centered counseling
		Peer review
	
	Standard educational materials	Extended educational materials

	----	Inviting PCPs during IDCT meetings to discuss patient cases
	
	----	Providing structured communication forms to PCPs by IDCT
	
	----	Distribution of shared care protocol + referral indication

**Feed-back**	At start and end of project: summary of clinical performance	Every 3 months: summaries of clinical performance
	
	----	Every three months: benchmarking feed-back

**Reminders**	Clinical reminders at start and end of project	Every three months: Clinical reminders
	
	----	Every three months: Shared care reminders

**Table 4:** Interventions for UQIP versus AQIP (organisational).

**ORGANISATIONAL**		
	**USUAL QUALITY IMPROVEMENT PROGRAM (UQIP)**	**ADVANCED QUALITY IMPROVEMENT PROGRAM (AQIP)**

**Team changes**	Interdisciplinary Diabetes Care Team (IDCT) operating close to regular care	Active installment of Interdisciplinary Diabetes Care Team (IDCT) operating under supervision of a diabetologist from a University Hospital
		
		Diabetes Program manager providing logistic support to PCPs
	
	----	Introduction of shared care protocol
		Active encouragement by IDCT and scientific team of PCPs to use shared care protocol
	
	----	Referral arrangements Active encouragement by IDCT and scientific team to adhere to referral arrangements
	
	----	Liaison activities by IDCT towards in-hospital diabetes care team in secondary care
	
	----	Involvement of independent pharmacists

**Continuous quality improvement**	Quality Assurance Team	Quality Assurance Team

The IDCT acted as a cornerstone to both the intervention arms, but the number, type and intensity of IDCT related interventions varied depending upon the intervention arm. The IDCT was installed at two locations in a physician-led primary care center that was well known and approachable to both PCPs and patients. A primary goal of the IDCT was to support the physicians in the co-management of diabetes. The IDCT included a nurse educator, an internist, a dietician and an ophthalmologist. The team provided individual patient counseling, didactic goal setting and situational problem solving as key educational methods to both the intervention arms. A health psychologist was only available to physicians of the AQIP, as well as a traveling educator providing education in the physician's practice or at the patient's home. Other interventions that were only provided to the AQIP were group educational sessions and the provision of structured educational materials for patients.

The IDCT was actively supervised by a diabetologist and bi-weekly interdisciplinary meetings were organized between the members of the team. Only physicians of the AQIP were invited to the latter meetings to discuss complex patient conditions. The IDCT met their colleagues from the university hospital-based diabetes team on a three-monthly basis to exchange experiences and to discuss complex patient cases. In addition, the internists of the IDCT met the supervising diabetologist on a two-monthly basis to discuss individual patient cases. Physicians of the AQIP were actively encouraged on a three-monthly basis to make use of the IDCT. All patient related interventions of the IDCT were registered. Interventions included consultations, telephone calls between a member of the team and the GP, email and written reports.

### Variables

Dependent variables related to the first study hypothesis on the feasibility of the IDCT were the use of the IDCT in both physicians and patients for all caregivers of the IDCT. The independent variable was the type of intervention arm. The expected participation rate in physicians was set at 80% whilst that in patients was set at 30%. International studies have shown that 30-70% of the patients with type 2 DM in primary care settings are not at target [27-29] of which some patients might benefit from services provided by an interdisciplinary team. Since PCPs currently provide 80% to 95% of diabetes care in Belgium, which includes a broad range of medical, educational, and psycho-social interventions in the management of diabetes, we estimated that 30% of the patient population in our project would benefit from co-management with a diabetes team.

For the second study hypothesis on the effectiveness of the IDCT, when comparing users and non-users, the dependent variables were the proportion of patients reaching three clinical ADA targets: (1) glycated hemoglobin (HbA1c) < 7%; (2) systolic blood pressure (SBD) ≤ 130 mmHg; and (3) low density lipid cholesterol (LDL-C) < 100 mg/dl. Other dependent variables were the mean improvements in individual values of 12 validated parameters: HbA1c, LDL-C, SBP, diastolic blood pressure (DBP), high density lipid cholesterol (HDL-C), total cholesterol (Tot. chol), weight, physical exercise, healthy diet, smoking status, statin and anti-platelet therapy and the proportion of patients reaching ADA targets. Independent variables were the use or either non-use of the IDCT in all patients. For the third hypothesis on the effectiveness of the IDCT when comparing users of the IDCT in both the intervention arms (AQIP and UQIP) the dependent variables were the same as for the second research hypothesis. The independent variable was the type of intervention arm.

Use of the IDCT was defined as having at least one consultation with a member of the IDCT.

### Statistical analysis

Baseline patient characteristics of IDCT users and non-users were compared with t-tests for continuous and χ^2^-tests for dichotomous outcomes, respectively. Endpoints were analyzed according to the intent-to-treat approach. Means and standard deviations were reported for continuous outcomes, and proportions for dichotomous variables. In order to study the evolution of the various outcomes over time, and to study how those evolutions depend on patient and/or PCPs characteristics, including use of IDCT, mixed models with random patient effects were used, which correctly accounted for the clustered nature of the repeated measurements within subjects. Continuous outcomes were analyzed with linear mixed models. Dichotomous outcomes were analyzed using logistic mixed models. In the latter case, model fitting was based on Gaussian quadrature with 200 quadrature points. In order to test for differences between both intervention arms, in the effects of IDCT use on patient outcomes, multiple linear and logistic regression models were used, whenever appropriate. Covariates included the use of IDCT, intervention and their interaction. Note that mixed models implicitly correct for baseline differences between patients when comparing the intervention arms. Alternatively, models could have been used which include the baseline outcome values as covariates. A comparison of several methods for correction for baseline differences has been described by Verbeke and colleagues [30]. Recent research has shown that mixed models, as applied in our analyses, have important advantages over other models that include baseline values as covariates [31].

As this paper is exploratory in nature, where many potential effects were tested, no correction for multiple testing has been performed. Effects that were found to be promising can be investigated in future studies. All analyses were performed using SAS, version 9.

## Results

### Participant flow

**Figure 1:**
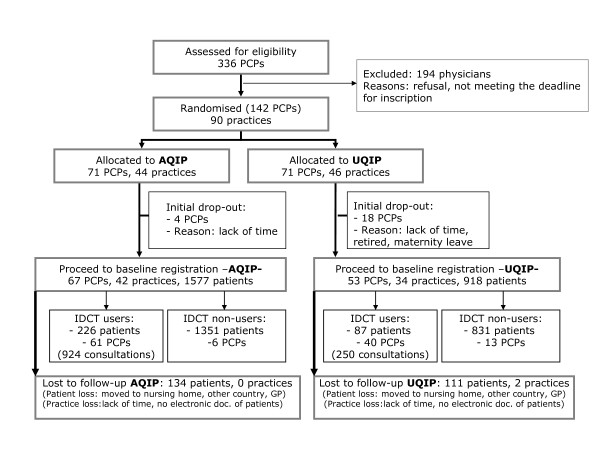
**Flowchart displaying Enrollment of patients and physicians in the study**.

Enrollment of physicians and patients in the study is presented in figure 1.

120 primary care physicians registered baseline data from 2495 patients (AQIP: 67 physicians, 1577 patients; UQIP 53 physicians, 918 patients). During the 18-month intervention period 2 physicians and 239 patients dropped out. Final results refer to 67 physicians, 42 practices and 1449 patients from the AQIP and 51 physicians, 32 practices and 807 patients from the UQIP.

### Overall results

After 18 months of intervention a significant improvement in all variables was noted (p < 0.0001), irrespective of the intervention arm. When comparing the two intervention arms (AQIP and UQIP) no significant differences in outcomes were noted after 18 months of intervention. A detailed analysis on the evolution of primary and secondary outcomes in all patients and for both the intervention arms will be provided in a separate article.

### Baseline characteristics of patients

Baseline information in users and non-users of the IDCT and for AQIP and UQIP is presented in table 5 and 6 respectively. Data show that users of the IDCT were younger (p < 0.0001), represented more men (p = 0.0001) and were more highly educated compared to non-users of the IDCT (p = 0.0002). Mean HbA1C was significantly higher in users of the IDCT (p < 0.0001), as well as mean DBP (p = 0.0002) and total cholesterol levels (p = 0.0223). The proportion of patients with HbA1C levels <8% was significantly lower in IDCT users compared to non-users (p < 0.0001). The use of metformin in case of obesity was significantly higher in users of the IDCT compared to non-users of the IDCT (p = 0.0008).

**Table 5:** Baseline information in users and non-users of the IDCT.

	**IDCT users (n = 313)** **Mean (SD)**	**IDCT non-users (n = 2182)** **Mean (SD)**	**p**
Mean age (years)	62.3 (11.5)	68.5 (11.6)	<0.0001

Mean diabetes duration (years)	6.5 (6.7)	7.3 (7.1)	0.0674

Female gender (%)	46	52	0.0001

HbA1c (%)	7.8 (1.6)	7.1 (1.2)	<0.0001

SBP (mm Hg)	136 (16)	136 (16)	0.8012

DBP (mm Hg)	81.2 (8.6)	79.3 (8.9)	0.0002

T. Chol (mg/dl)	197 (39)	191 (41)	0.0223

LDL-C (mg/dl)	111 (32)	108 (34)	0.1181

HDL-C (mg/dl)	53 (16)	54 (15)	0.4763

BMI	30.4 (5.3)	29.5 (5.3)	0.0087

Duration of insulin therapy	6.2 (7.3)	7.9 (7.6)	0.1820

*Education level*			

Low education level (%)	40	50	0.0002

High education level (%)	20	17	0.0669

*Proportion of patients with:*			

HbA1c < 8%	62	84	<0.0001

HbA1c < 7%	37	57	<0.0001

SBP ≤ 130 (mm Hg)	48	50	0.4892

LDL-C < 100 (mg/dl)	36	42	0.0621

Non smoker (%)	88	85	0.3332

Healthy Diet (%)	63	67	0.1201

Physical Exercise (%)	59	52	0.3597

Aspirin/clopidogrel (%)	35	37	0.0952

ACE/A2A treatment (%)	31	34	0.1945

Statin (%)	37	40	0.3424

Metformin if obesity (%)	71	58	0.0008

Insulin (%)	16	20	0.1063

Complications (microangiopathic)	86	71	0.1351

**Table 6:** Baseline information in users of the IDCT for UQIP and AQIP.

	**Users of IDCT (AQIP)** **(n = 226)** **Mean (SD)**	**Users of the IDCT (UQIP)** **(n = 87)** **Mean (SD)**	**p**
HbA1c (%)	7,72 (1.62)	7,98 (1.59)	0.2632

SBP (mm Hg)	135,35 (15.28)	138,85 (19.09)	0.1752

DBP (mm Hg)	80,69 (8.52)	82,62 (8.72)	0.3202

TCHoL (mg/dl)	193,96 (39.51)	204,01 (38.25)	0.0494

LDL CHol (mg/dl)	108,94 (32.56)	115,19 (31.09)	0.2304

HDL CHol (mg/dl)	53,43 (15.83)	53.51 (15.61)	0.9089

BMI	30,49 (5.22)	30,17 (5.52)	0.5399

Targets reached (%)	85	75	0.1324

Smokers (%)	88,35	85,9	0.5571

Healthy diet (%)	62,36	63,89	0.8570

Physical exercise (%)	55,07	69,23	0.0477

Aspirin/clopidogrel (%)	36,56	37,21	0.7233

ACE/A2A treatment (%)	68,72	65,12	0.4532

Statin treatment (%)	40,09	27,91	0.0391

Users of the IDCT in the UQIP had worse levels of primary and secondary endpoints at baseline compared to users of the IDCT in AQIP. No significant differences in outcome measures were found at baseline when comparing UQIP and AQIP[32]. Specific baseline data on comorbidity revealed no significant differences between users and non users of the IDCT in both the intervention arms, and irrespective of the intervention arm. Comorbidity was pre-defined as a reported history of heart attack, cerebrovascular disease, micro-angiopathic complications, history of angina, hypertension, dyslipidemia, PAD or coronary, cerebral or peripheral vascular intervention in the personal history and depression.

### Use of IDCT in PCPs and patients

A total of 101 PCPs (84%) and 313 patients (12.5%) made use of the IDCT. More male PCPs (55%) referred patients to the IDCT compared to their female peers (45%). PCPs who worked together in the same practice also made more use of the IDCT compared to PCPs that did not use the IDCT (63% and 58% respectively).

When comparing the two intervention arms the use of the IDCT in PCPs and patients was significantly higher in the AQIP compared to the UQIP (AQIP = 61 PCPs (91%); UQIP = 40 PCPs (75%); p = 0.02) and (AQIP = 226 patients (14.3%), vs. UQIP = 87 patients (9,4%); p = 0.03), respectively. The number of patients per practice that were referred to the IDCT ranged from 0-15 in the AQIP and from 0-16 in the UQIP. An overview on the IDCT use in both UQIP and AQIP is presented in table 7. Referral subgroups in AQIP included 138 (8,7%) patients which consulted the dietician, 107 (6,8%) patients which consulted the educator, and 79 (5,0%) patients which consulted the internist. The 'travelling educator' was consulted by 40 patients (2,5%) and the health psychologist by 18 patients (1,1%). Referral subgroups in UQIP for the dietician, educator and internist included 40 (4,3%), 38 (4,1%) and 29 (3,1%) patients respectively. Patients in the AQIP represented 79% of all consultations with the IDCT (AQIP = 924; UQIP = 250). In AQIP the mean consultation rate per patient was 4 and in UQIP 3.

**Table 7:** Use of IDCT services by PCPs and patients in UQIP and AQIP.

**Type of service offered by IDCT**	**UQIP**	**AQIP**
IDCT consultations	87 patients (9.5%) referred by 40 PCPs (75%) 250 consultations (21%)	226 patients (14.3%) referred by 61 PCPs (91%) 924 consultations (79%)
Educator in primary care facility	38 patients (4.1%) 94 consultations	107 patients (6.8%) 256 consultations
Educator at home or in PCP practice	NA	40 patients (2.5%) 91 consultations
Dietician	40 patients (4.3%) 63 consultations	138 patients (8.7%) 255 consultations
Internal medical doctor	29 patients (3.1%) 63 consultations	79 patients (5.0%) 164 consultations
Opthalmologist	19 patients (2.1%) 30 consultations	55 patients (3.5%) 85 consultations
Health psychologist	NA	18 patients (1.1%) 73 consultations
Printed educational materials for patients	NA	126 distributed
Communication forms to PCPs	NA	924 reports
Free blood monitoring tools for patients with insulin therapy onset	NA	107 distributed
Group information sessions for patient and family	NA	7 sessions, 310 participants from 14 physicians

After 18 months of intervention, 27% (n = 598) of the total patient population did not reach the HbA1c target and were not referred to the IDCT.

Patients with an HbA1c level between 7% and 8% made more use of the IDCT compared to patients with HbA1c levels < 7% (Odds Ratio = 2.0, p = 0.0003). The Odds Ratio (OR) in patients with HbA1c >8% compared to patients with an HbA1c < 7% was 3.0 (p < 0.0001). The OR in patients with an LDL level between 100-115 mg/dl compared to patients with a level <100 mg/dl was 1.4 (p = 0.038). The OR in patients with an LDL level ≥ 115 mg/dl was 1.5 compared to patients with an LDL level < 100 mg/dl (p = 0.0023). The OR in patients with insulin initiation during the project was 5.5 compared to patients without insulin initiation (p < 0.0001).

### Outcomes in users and non-users of the IDCT

**Figure 2:**
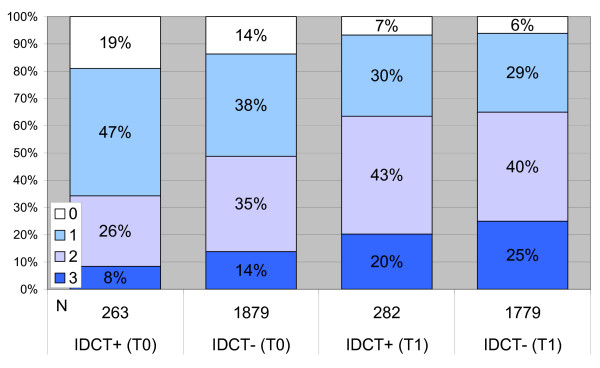
**Proportions of patients reaching therapeutic targets in users and non-users of the IDCT**. N = total number of included patients in the different subgroups. IDCT+ = group of patients who have consulted the IDCT and who presented with values of all three primary outcome parameters. IDCT- = group of patients who have not consulted the IDCT and who presented with values of all three primary outcome parameters.

After 18 months of intervention the use of the IDCT was significantly associated with improvements in HbA1c (p = 0.00001) and LDL-cholesterol (p = 0.00039) and an increase in both the use of statins (p = 0.04308; OR: 1.902) and anti-platelet therapy (p = 0.00544; OR: 2.213). A detailed overview of the evolution in all parameters and results for IDCT users and non-users is provided in table 8. As presented in figure 2. IDCT use was also significantly associated with the number of targets that was reached (p = 0.005).

**Table 8:** Evolution of variables in all patients (T0: baseline value, T1: value after 18 months of intervention) and IDCT use/non-use.

	**T0** **120 physicians, 67 practices** **2495 patients** **Mean (SD)**	**T1** **118 physicians, 65 practices** **2256 patients** **Mean (SD)**	**Difference between T1-T0**	**P**
HbA1c (%)	7.15 (1.26)	6.76 (0.95)	-0.39	<0.0001

IDCT users	7.78 (1.63)	7.00 (1.09)	-0.78	<0.0001
		
IDCT non-users	7.05 (1.16)	6.72 (0.92)	-0.33	

SBD (mm Hg)	136 (16)	133 (15)	-3	<0.0001

IDCT users	136 (16)	133 (15)	-3	0.6335
		
IDCT non-users	136 (16)	133 (15)	-3	

DBD (mm Hg)	79 (9)	77 (9)	-2	<0.0001

IDCT users	81 (9)	79 (9)	-2	0.6103
		
IDCT non-users	79 (9)	77 (9)	-2	

Tchol (mg/dl)	192 (40)	177 (37)	-5	<0.0001

IDCT users	196 (39)	173 (38)	-23	0.0002
		
IDCT non-users	192 (41)	177 (37)	-15	

HDL-C (mg/dl)	54 (16)	55 (15)	+1	0.0006

IDCT users	54 (16)	55 (16)	+1	0.8242
		
IDCT non-users	54 (15)	55 (15)	+1	

LDL-C (mg/dl)	108 (34)	95 (32)	-13	<0,0001

IDCT users	110 (32)	90 (33)	-20	0.0012
		
IDCT non-users	108 (34)	95 (32)	-13	

BMI (kg/m^2^)	29.6 (5.3)	29.3 (5.2)	-0.3	<0,0001

IDCT users	30.3 (5.4)	30.0 (5.3)	-0.3	0.9737
		
IDCT non-users	29.5 (5.2)	29.2 (5.2)	-0.3	

Hba1c < 7%,	54%	67%	+13%	<0.0001

IDCT users	37%	57%	+20%	0.07449
		
IDCT non-users	57%	69%	+12%	

BMI < 25 kg/m^2^	18%	20%	+2%	0.00108

IDCT users	15%	18%	+3%	0.7186
		
IDCT non-users	19%	21%	+2%	

Non smokers	86%	89%	+3%	0.023

IDCT users	88%	89%	+1%	0.2675
		
IDCT non-users	85%	89%	+4%	

Healthy nutrition	67%	75%	+8%	<0.0001

IDCT users	63%	77%	+ 4%	0.9885
		
IDCT non-users	67%	75%	+8%	

Physical exercise	53%	60%	+7%	0.00035

IDCT users	59%	71%	+12%	0.3349
		
IDCT non-users	52%	59%	+7%	

Aspirin/clopidogrel	40%	57%	+17%	<0.0001

IDCT users	37%	67%	+30%	0.005644
		
IDCT non-users	40%	56%	+16%	

ACE/A2A	73%	78%	+5%	<0.0001

IDCT users	68%	76%	+8%	0.3954
		
IDCT non-users	74%	78%	+4%	

Statins	39%	53%	+14%	<0.0001

IDCT users	37%	57%	+20%	0.04431
		
IDCT non-users	40%	53%	+13%	

Outcomes in users of the IDCT in AQIP and UQIP

When comparing the two intervention arms with respect to the effects of IDCT use on patient outcomes, a significant difference was obtained for the use of anti-platelet therapy only (Table 9).

**Table 9:** Effect of IDCT use in AQIP compared to UQIP*.

	**Estimate**	**StdErr**	**P-value**
HbA1c (%)	0.0848	0.1476	0.5656

SBP (mm Hg)	2.3901	2.2566	0.2896

DBP (mm Hg)	0.4532	1.3308	0.7335

T. Chol (mg/dl)	1.8074	5.379	0.7369

LDL-CL (mg/dl)	0.1833	4.686	0.9688

HDL-C (mg/dl)	-1.4947	1.4404	0.2995

BMI	0.0092	0.2799	0.9737

Targets (%)	-0.3404	0.4078	0.4039

Non smoker (%)	1.0710	1.3691	0.4341

Health diet (%)	0.4817	0.6984	0.4904

Physical exercise (%)	1.0129	0.6550	0.1221

Aspirin/clopidogrel (%)	1.3368	0.5702	0.0119

ACE/A2A treatment (%)	1.4285	0.7558	0.0584

Statin treatment (%)	0.2726	0.6341	0.1766

* Reported estimates represent the difference of the effect of IDCT use on each outcome in the AQIP group and the effect of IDCT use in the UQIP group.

## Discussion

The results of this trial demonstrate that the use of interdisciplinary diabetes care teams which are actively guided and supported by a specialist team from secondary care are associated with important improvements in outcomes of care. Another important finding of our study is that the high number of physicians that made use of these teams demonstrates that the implementation of interdisciplinary shared care in a health care setting with limited tradition in shared care is feasible. The expected participation rate of 80% in physicians was easily attained, with physicians in the AQIP to make more use of the IDCT compared to physicians of the UQIP. The latter is possibly explained since physicians in the AQIP were actively encouraged by the program manager to make use of the IDCT whereas physicians in the UQIP were not. These findings highlight the need for specific implementation strategies in settings that have limited tradition in shared care. The higher referral rate in AQIP was however not associated with better patient outcomes which possibly indicates that these differences were not large enough to induce a significant effect. The relative absence of clinical significant differences between users of the IDCT when comparing the two intervention arms might also be related to the limited use of supplementary interventions as provided to physicians and patients of the AQIP. For example, very few physicians or their patients consulted the health psychologist or the travelling educator. These service offerings were relatively unknown to physicians and patients prior to the start of the project. Another possible reason for the aforementioned finding is that patients in the UQIP that used the IDCT had significantly worse levels in their outcomes at baseline that allowed for greater improvements over the 18-month intervention period.

In contrast to the overall participation rate of physicians, patients less often made use of the IDCT (12,5%) which means the expected participation rate of 30% in patients was not attained. It is not clear to what extent this is related to either physician referral patterns, patient behaviours or both. Physicians generally refer to a diabetes care team once their threshold for comfort with diabetes management has been surpassed [33]. In our study higher levels of HbA1c, LDL-cholesterol and insulin initiation indeed seemed important triggers for referral to the IDCT. As physicians in our study were recommended to refer to the IDCT in case the treatment targets were not met, they have focused on those patients that showed an important need for interdisciplinary shared care. Data show however that a substantial proportion of the patients who did not reach treatment targets did not make use of the IDCT. This might in part be explained by clinical inertia in physicians, but it is reasonable to assume that physicians had difficulty to motivate their patients to consult the IDCT despite their own convictions on the added value of the team in the co-management of diabetes. More than 95% of diabetes care is done by the patient, and health professionals have very little control over how patients manage their illness between office visits. Patients manage their diabetes on a daily basis within the context of the other goals, priorities, health issues, family demands, and other personal concerns that make up their lives[34]. Previous studies have shown that patients with diabetes often show poor compliance with diet, exercise, medication regimen and even appointment compliance[35]. These behaviours are in their turn affected by numerous variables, such as the nature of the patient-physician relationship, complexity of regimen, disruption of lifestyle, emotional support, financial resources, education in self-management skills, cues to action, perceived barriers, locus of control and motivation[36]. In our study patients who were not at target might have been unwilling to consult the IDCT, despite recommendations of their PCPs. These findings highlight the need for ongoing patient empowerment strategies in the context of the physician-patient relationship as well as clear referral indications in order to prevent over and under use of interdisciplinary shared care services in both physicians and patients. The broad referral indication that was applied in our project was probably not specific enough to guide PCPs in their decision to refer patients to the IDCT. The American Diabetes Association (ADA) standards currently do not indicate a minimum frequency for the provision of diabetes education, including dietary advice[4]. Other and clearer referral criteria for specialist services defined by the ADA are e.g. recurrent hypoglycaemia, poor glycaemic control and persistent hypertension and/or hyperlipidaemia despite intensive management and the need for psychosocial/counselling support to overcome barriers to self-care.

As an explanation to the positive results that are associated with the use of the IDCT we assume that particular team characteristics such as quality task orientation and shared leadership with clearly defined and valued group goals that the teams were trained at prior to the intervention might possibly have contributed to the success of the team. There is an extensive basis of research demonstrating the aforementioned team characteristics are associated with improved outcomes of care [37-39].

Limitations to our study design are twofold. The UK Medical Research Council (MRC) Framework that we used for the development of our complex intervention demonstrated the difficulty of balancing optimum study design with designing interventions that were practical enough to be applied in family practice. The MRC Framework provides a methodological rather than an explanatory approach to evaluating complex interventions. This means that in order to fully understand and predict problems of workability and integration of complex interventions the additional use of psychological and sociological models is important in the design and evaluation of complex interventions. The Normalization Process Model [40] is a recent sociological model that asks what people *do *to make a complex intervention workable, and to integrate it in practice. The model proposes that complex interventions are implemented in processes in which the collective action and interaction of patients, professionals and others are governed by four factors. These factors are interactional and skill-set workability and relational and contextual integration. Since we did not make use of such additional social and psychological models in the design of our program, we recognize this is a limitation to our study protocol. We also have not integrated qualitative methods within the context of a pilot trial that could have helped to interpret the quantitative results of the pilot trials by clarifying process and outcome results. What we consider a particular strength of the study is it provides answers to hypotheses on innovative types of change interventions (such as interdisciplinary teams operating on the primary-specialty care interface). These hypotheses were tested using a large group of physicians and patients during an 18-month period. Most quality improvement programs reduce the intervention period to six months and are limited to smaller groups. A six-month period is short considering the Hawthorne effect that probably has not been washed out. Another strength of our study is that the primary and secondary care interface is strongly valued since we recognize a well-operationalized interface as an important attribute to high quality diabetes care [41]. More specific, the clinical leadership and coaching provided by a diabetologist to both the primary care physicians and the interdisciplinary diabetes care team is of particular importance in fragmented health care systems including the one from Belgium. Another strength of the study is the explicit project focus on multiple cardiovascular risk factors. A systematic review published in 2001 observed that most interventions did not pay enough attention to patient outcomes and if so, only changes in glycemic control were evaluated [42]. A last strength of the study is the use of all six dimensions of the Chronic Care Model (CCM), which is to our knowledge the fourth study in the field of diabetes care to do so [43-45]. The use of all six dimensions of the CCM allows for an evaluation how some of the CCM components are associated with improved outcomes and thus it provides evidence to support the validity of this model.

Future research that will complement this present study will include 1. a qualitative analysis on perceived barriers to high quality diabetes through PCPs eyes, 2. a quantitative study on how patients' experiences with perceived quality of patient-centered care and self-management support are associated with documented diabetes outcome of care indicators and 3. a health economic assessment of the cost-effectiveness of the trial.

Overall, we assume that the increasing prevalence of diabetes, combined with its complexity, rapidly evolving medical therapies and the requirement for patient self-management will lead to a growing demand for multifaceted and interdisciplinary shared care services that target improvements in diabetes care[46].

## Conclusion

Interdisciplinary care teams that are part of a multifaceted quality improvement program are associated with improved outcomes of care. More research is however required on what type of team and program characteristics contribute to improvements in diabetes care. There is a particular need for clear referral indications in order to prevent over and underuse of interdisciplinary care services in persons with type 2 DM.

## Competing interests

The authors declare that they have no competing interests.

## Authors' contributions

LB, GG and CB participated in the study design and drafted the manuscript.

CM, BA, GV, AC, AI, JH and RG participated in the study design.

All authors have read and approved the final manuscript.

## Pre-publication history

The pre-publication history for this paper can be accessed here:


